# The Molecular Mechanisms of the Antibacterial Effect of Picosecond Laser Generated Silver Nanoparticles and Their Toxicity to Human Cells

**DOI:** 10.1371/journal.pone.0160078

**Published:** 2016-08-30

**Authors:** Peri Korshed, Lin Li, Zhu Liu, Tao Wang

**Affiliations:** 1 Faculty of Medical and Human Science, the University of Manchester, Manchester, M13 9PL, United Kingdom; 2 Laser Processing Research Centre, School of Mechanical, Aerospace and Civil Engineering, the University of Manchester, Manchester, M60 IQD, United Kingdom; 3 Corrosion and Protection Centre, the Mill, School of Materials, The University of Manchester, Manchester, M13 9PL, United Kingdom; Harbin Institute of Technology, CHINA

## Abstract

Silver nanoparticles (Ag NPs) are known to have antibacterial properties. They are commonly produced by chemical synthesis which involves the use of harmful reducing agents. Contras, the laser technique is able to generate high-purity Ag NPs in water with specified surface charge characteristics. In the past, the molecular mechanisms contributing to the bactericidal effects of Ag NPs have been investigated extensively, but little is known of the antibacterial and toxic effects and mechanisms involved in laser-generated Ag NPs. In the current study Ag NPs were generated by picosecond laser ablation. Their antibacterial activity was determined on the gram-negative bacteria *E*. *coli* and *Pseudomonas aeruginosa*, and the gram positive bacteria *Staphylococcus aureus* including the methicillin resistant strain MRSA. Results showed that the laser generated Ag NPs exhibited strong dose-dependent antibacterial activity against all the three bacterial strains tested. Using *E*.*coli* as a model system, the laser Ag NPs treatment induced significantly high levels of reactive oxygen species (ROS). These ROS did not include detectable hydroxyl radicals, suggesting for the first time the selective ROS induction in bacterial cells by laser generated Ag NPs. The increased ROS was accompanied by significantly reduced cellular glutathione, and increased lipid peroxidation and permeability, suggesting ROS related bacterial cell damage. The laser generated Ag NPs exhibited low toxicity (within 72 hours) to five types of human cells although a weak significant decrease in cell survival was observed for endothelial cells and the lung cells. We conclude that picosecond laser generated Ag NPs have a broad spectrum of antibacterial effects against microbes including MRSA with minimal human cell toxicity. The oxidative stress is likely the key mechanism underlying the bactericidal effect, which leads to lipid peroxidation, depletion of glutathione, DNA damages and eventual disintegration of the cell membrane.

## Introduction

The fast appearance of new bacterial strains resistant to current available antibiotics has become a growing obstacle to public healthcare. Almost 5,000 hospital deaths per year were caused by multi-drug resistant bacteria (MDR) such as methicillin resistant *Staphylococcus aureus* (MRSA) in the UK alone [1]. This increase in the pathogenic bacterial resistance to drugs motivated the search for new antimicrobial therapeutic agents [2], and nanoparticles (NPs) are considered to be good candidates for this purpose. The unique physical and chemical properties of NPs associating to their ability to inhibit microbial growth or kill microbes led to increased research in order to enhance their antibacterial efficacy and specificity, reduce their toxicity to human cells, and understand the mechanisms behind their actions. In the past decade numerous types of NPs have been developed for antibacterial applications. Although efforts have largely been devoted to the development of drug carrier platforms using mainly organic NPs, metallic NPs still stand out as promising therapeutic agents due to their direct antimicrobial activities. Several metallic NPs including silver (Ag), copper (Cu), titanium (Ti), Zinc (Zn), and their oxide derivatives were identified to exhibit antimicrobial effects, among which Ag NPs are the most popular and widely used in both clinical practice and domestic consumables [3,4].

Silver has historically been widely known to have antibacterial properties [5]. Nano-silver greatly enhances the functionality of the material and it has been used in diverse healthcare applications including but not limited to wound dressings, bandages, ointments, lotions, water purifications and medical devices [6] as well as protective agents for patients with HIV [7]. Most importantly, Ag NPs have a broad spectrum of antibacterial qualities against a wide range of gram-positive and gram-negative bacteria and do not contribute to the development of resistance strains. Therefore, Ag NPs have the potential to be widely used against drug resistance bacteria such as MRSA. It is estimated that, as an antimicrobial agent, silver nanoparticles have an annual demand of 3,125 tons/year for medicine and 2,800 tons/year in the field of food, hygiene and water purification [8].

Metallic NPs can be produced in several different ways including physical, chemical, and biological methods [9]. Despite successful applications, most of the methods are still expensive owing to the use of hazardous chemicals [10]. Laser ablation in aqueous phase is considered a unique technique that is simple and fast, and able to produce NPs in any desired concentration with high purity without relying on chemical reactions. Therefore the laser produced NPs are ideal for medical applications and environmentally friendly too [11]. Most importantly, the metallic NPs generated by laser ablation have different surface properties compared to their counterparts generated by chemical methods. One of the unique properties is the surface charge [12] which makes the NPs prone to interacting with the negatively charged bacterial surfaces and proteins, making them ideal for targeting microbes. Although the molecular mechanisms contributing to the bactericidal effects of Ag NPs have been investigated extensively in the past, there has not been sufficient documentation on laser-generated Ag NPs. To our knowledge, few publications have addressed the antibacterial effects of laser-generated Ag NPs and vary little information on the action mechanisms has been provided [13]. None of the information is on biological mechanisms and toxicity of picosecond laser generated Ag NPs.

The exact mechanisms through which Ag NPs exert their antibacterial effect are still under investigation. It was observed, that Ag NPs, by interacting with bacterial cell membranes through making pits and holes, accumulate inside bacterial cells where they bind to important cellular machineries, damaging cell function [14]. In addition to the physical interactions, the release of silver ion (Ag^+^) is considered as an important mechanism for the bactericidal effect of Ag NPs. The interaction between positively charged Ag^+^ and the negatively charged bacterial cell surface disrupt the selective permeability and structure of the bacterial cell membrane [15], resulting in protein leakages [16]. After having penetrated bacterial cell, Ag^+^ interacts with DNA and ribosomes resulting in DNA damage and influencing the protein translation. Ag^+^ also interacts with the thiol group of proteins and essential enzymes in the cellular respiratory chain, generating reactive oxygen species (ROS) such as superoxide anion (O_2_^-1^), hydroxyl radical (·OH) and singlet oxygen (^1^O_2_) leading to cell death. The oxidative damage caused by the ROS generation is considered a key mechanism that, synergistic with the effect of Ag^+^, underlies the bacterial killing activities of Ag NPs [17].

Despite the therapeutic potential, the use of Ag NPs has been largely limited to non-*in vivo* applications, such as medical dressings. This is mainly due to considerations of their potential toxicity to humans [17,18]. Numerous studies have demonstrated the toxicity of metal nanoparticles to human cells [19]. For example, a study by Asharani et al attributed the toxicity of Ag NPs on human lung epithelial cells to the formation of ROS which leads to oxidative stress and cell damage [20]. Paddle-Ledinek et al also demonstrated the cytotoxic effect of Ag NPs extracted from wound dressings (Acticoat) to human keratinocytes [21]. The cytotoxicity of Ag NPs were also determined on other cell types such as human fibrosarcoma cells [22] and the liver cells of rats [23] via mechanisms including DNA damage, apoptosis, depletion of antioxidant glutathione, reduction in the potential of mitochondria membrane and ROS formation. In addition, the absorption of Ag NPs into the blood from wound dressing was observed which led to argyria symptoms [24].

However, data provided by the published studies on the toxicity of Ag NPs to different mammalian cells had large variations. It was noticed that Ag NPs acted on both human and bacterial cells, but the toxicity to human cells was very low compared to that of bacterial cells when the same concentration was used [25]. This may be due to the differences in the cell membrane structures between mammalian and bacterial cells and the existence of endocytic machinery and multiple intracellular compartments in human cells which prevent the NPs from easily penetrating the membranes to interact directly with important molecules within the cells. The human immune system serves as an additional protective barrier for the clearance of NPs [26]. Additionally, the discrepancies seem also to rely on the manufacturing methods and the size of the NPs as well as the cell types used in the toxicity study. For these reasons there has been no general consensus concerning the toxicity of Ag NPs to human cells [27]. Furthermore, there has been a lack of information about the toxicity to humans of laser generated Ag NPs.

We recently produced Ag NPs using a picosecond laser ablation method [28]. In this study, we sought to conduct a comprehensive analysis on the antibacterial activities, the molecular mechanisms underlying their bactericidal effects, and the toxicity to human cells of the picosecond laser generated Ag NPs. We have demonstrated a broad spectrum of antibacterial activities for the laser generated Ag NPs on both gram-negative bacteria *E*. *coli* and *Pseudomonas aeruginosa*, and the gram-positive bacteria *Staphylococcus aureus* such as MRSA. We identified multiple molecular and cellular mechanisms that contribute to the bactericidal effects of laser Ag NPs, and determined their toxicity to different types of human cells originating from the lungs, skin, blood vessels, kidneys and the liver. We anticipate that our data would contribute to a better understanding of laser generated NPs in general and pave the way for future applications for laser generated Ag NPs in healthcare.

## Materials and Methods

### Nanoparticles production

Nanoparticle production by pulsed laser ablation was described in our previous publication [28]. Briefly, an Ag plate (dimensions of 25mm × 25mm × 2mm, purity 99.99%) was sterilised by immersing into the ethanol and then the autoclaved deionised water (dH_2_O). The Ag plate was then immersed in 20 ml of dH_2_O in a glass vessel. A picosecond pulsed Nd: YVO4 laser with a wavelength of 1064 nm was used to ablate the Ag plate at a high scanning speed at a pulse repetition rate of 200 kHz, beam spot size 125 μm and an average power of 9.12 W. The weight of the silver plate was measured using an analytical balance before and after laser ablation. The concentration of the generated Ag NPs was calculated by taking the difference of the two weight measurements and divided by the volume of dH2O in which the laser ablation was carried out, and presented as μg/ml.

### Bacteria culture and the determination of antibacterial activities of NPs

Bacterial strains *P*. *aeruginosa* (ATCC, 9027) [29] and methicillin resistant *S*. *aureus* (ATCC, 43300) [30] used in this study were kindly provided by the Medical Group Biology Services in the Faculty of Life Sciences, the University of Manchester. *E*.*coli* (JM 109) [28] was purchased from Promega and used in our previous study. A single colony of each type of bacterial was inoculate in 10 ml of autoclaved Muller-Hinton broth media (Sigma), respectively and incubated at 37°C overnight with shaking at 225 rpm. The culture of bacteria suspension was diluted to give 10^4^ cfu/ml ready to be used for the antibacterial experiments described below. The antibacterial activity of NPs was determined following the standard Nathan’s Agar Well Diffusion (NAWD) technique. Briefly, a lawn of each bacterial culture prepared above was spread uniformly on the Muller-Hinton agar plates using sterile cotton swabs and left for 10 minutes for culture absorption, and then multiple 6 mm wells were made by punching the Muller-Hinton agar plates using a cylinder glass tube. Fifty μl of NP sample was added into each well and was incubated at 37°C for 18 hours. The zones of inhibition (ZOI), which reflects the susceptibility of microbes to the NPs were then measured [31].

### Detection of ROS generation

Two types of ROS detection reagents, 2,7-dichlorofluorescein diacetate (DCFH-DA, Sigma-Aldrich) and Hydroxyphenyl fluorescein (HPF, Life Technologies), were used in the study. First of all, 100 μl of laser generated Ag NPs at different concentrations (10, 25 and 50 μg/ml) were incubated with 0.9 ml of bacterial culture suspension (10^4^ cfu/ml) as mentioned above for 5 hours at 37°C in triplicate with shaking at 225 rpm. The bacterial cells were then pelleted by centrifugation at 1000 rpm for 5–10 min. For the use of HPF reagent, the bacterial cell pellet was suspended in 1 ml of LB broth and mixed with 10μl of HPF to give a final concentration of HPF to be 50 μM. After incubating at 37°C for 1 hour in the dark, 200 μl of cell suspension was transferred to a well of a 96-well plate in triplicate. The ROS formation was measured by florescence spectrophotometer with a setting of excitation wavelength 530 nm and emission wavelength 515 nm. The florescence intensity correlates to the formation of ROS [32]. For the use of DCFH-DA kit, the bacterial cell pellet was suspended in a LB broth. DCFH-DA reagent was added to the cell suspension to give a final concentration of 100 μM followed by incubating at 37°C for 30 minutes in the dark. The fluorescence was measured as above at an excitation wavelength 485 nm and emission wavelength 528 nm [33].

### Lactate dehydrogenase (LDH) release assay

LDH catalyses the conversion of lactate to pyruvate via reduction of NADP to NADPH, which is associated with the formation of tetrazolium giving the red colour. The release of LDH was determined using the LDH assay kit (Fisher Scientific/ Cat No. 88954). Briefly, bacterial cells were cultured in 96-well plates (10^4^ cfu/ml) in the Muller-Hinton media in triplicate at 37°C overnight. The cells were then treated with 10 μl laser Ag NPs or 10 μl dH_2_O as control in a final concentrations of NPs to be 2–10 μg/ml and further incubated at 37°C overnight. Ten μl of lysis buffer was then added and mixed by gently tapping and incubated at 37°C for 45 minutes. Fifty μl cell lysate from each sample were then transferred to a new 96-well plate and 50 μl reaction buffer was added to each well and mixed gently. The plate was then left at room temperature for 30 minutes in dark before 50 μl stop solution was added to each well and mixed by gentle tapping. Finally, optical density (OD, Absorbance) was measured at 490 nm using a spectrophotometer (Omega).

### Protein leakage determination

The Bradford assay has been used to detect protein leakage using the kit provided by the manufacture (Thermo Scientific/ Cat No. 23200). One ml of *E*. *coli* (10^4^ cfu/ml) was mixed with 100 μl laser generated Ag NPs to give final concentrations ranging 5–20 μg/ml and incubated for overnight. One ml of the bacteria-Ag NP mix was centrifuged at 12,000 rpm for 10 minutes. Then 10 μl supernatant from each sample were transferred to 96 well plates followed by adding 250 μl of Coomassi Blue reagent. After mixing the plate on a plate shaker for 30 minutes and further incubation at room temperature for 10 minutes, the absorbance was measured at 595 nm using a spectrophotometer (Omega). BSA was used as the standard for which a standard curve was drawn at each experiment to determine the protein concentration for each sample.

### Glutathione reductase assay

The depletion in glutathione level was measured using the Glutathione reductase kit (GRSA, from Sigma). The assay principles include the reduction of oxidized glutathione by glutathione reductase enzyme. In turn, NADPH reduces to NADP causing decrease in absorbance at 412 nm followed by the appearance of a yellow by-product that is measured by optical density, which is proportional to the activity of glutathione reductase in the sample. Bacterial culture (10^4^ cfu/ml) was treated with 15 μg/ml laser generated Ag NPs by incubating in the shaker for 3–5 hours. H_2_O_2_ (4 ug/ml) was used as positive control, and equal volume of dH_2_O was used as negative (NP-free) control. Following the treatment of *E*. *coli* by Ag NPs, the glutathione reductase level was measured according to the instruction by the kit manufacture.

### Effect of lipid peroxidation (LPO)

Malondialdehyde (MDA) is a by-product of lipid peroxidation of bacterial cell membrane, as a marker of lipid peroxidation. MDA, reacts with thiobarbituric (TBA) form MDA-TBA complex (pink colour) which can be detected by the lipid peroxidation MDA assay Kit (Cat. No. MAK085, Sigma-Aldrich). Bacterial culture (10^4^ cfu/ml) was treated with different concentrations of laser generated Ag NPs (5, 10, 15 and 20 μg/ml) by incubating in the shaker for 3 hours. One ml of the bacterial suspension was homogenized in ice with 300 μl MDA lysis buffer and 3 μl of butylated hydroxytoluene BHT were used to distinguish between pigments formation due to degradation of lipophilic peroxides and those newly formed during the oxidative stress, then mixed and centrifuged at 13,000 g for 10 minutes to remove insoluble materials. Then 200 μl of the supernatant from each homogenized sample were placed into a microcentrifuge tube and 600 μl of the TBA solution were added into each tube. The reaction was incubated at 95°C for 60 minutes in order for the formation of MDA-TBA, and then cooled down by placing into ice bath for 10 minutes. Finally, 200 μl reaction mix from each tube were transferred into a well of 96-well plates for absorbance measurement at a wavelength of 532 nm.

### DNA fragmentation

Five ml of *E*. *coli* culture at density of 10^4^ cfu/ml were mixed with 1 ml of either laser Ag NPs 50 μg/ml or the commercial Ag NPs 20 μg/ml (Sigma) to give a final concentration to be 10 and 4 ug/ml respectively. The Ag NP-loaded bacterial culture was incubated at 37 C° with constant shacking at 225 rpm for 3 hours. The bacterial cells were then pelleted by centrifugation at 8, 000 ×g for 5 minutes, and the bacterial DNA was extracted using the Genomic DNA kit (Cat No. BioLine) according to instructions by the manufacture. 200 ng of DNA were loaded onto 1% agarose gel in TAE buffer, and electrophoresis was carried out at constant Voltage at 100V for 1.5 hrs.

### UV-Visible absorbent spectrum measurement

The absorbent spectrum of different concentrations of laser generated Ag NPs was measured by Synergy HTX Multi-Mode Reader (BioTek). Triplicate samples were measured at each Ag NP concentration.

### Zeta potential measurement

Zeta potential of the colloidal Ag NPs in water was measured with a ZetasizerNano ZEN3600 (Malvern Instruments) according to manufacturer’s instruction. Three readings were automatically taken by the instrument and standard deviation (St Dev) was calculated by the building-in software.

### Silver ion measurement

The silver ion Ag+ released from the Ag NPs was measured by Inductively Coupled Plasma Mass Spectrometer (ICP-MS, Agilent 7500cx). Samples of Ag NPs were dilute in 2% HNO_3_. The instrument was first calibrated and ran a blank containing 2% HNO_3_ and then standards ranging from 1, 2, 5, 10 and 50 ppb Ag measuring the 107 Ag isotope. The instrument then read back the concentration it had determined to verify the calibration. A wash by 2% HNO_3_ was carried out to wash out any Ag left in the instrument from the 50ppb standard and then ran a different Ag containing standard to ensure the measurement on the calibration standards are accurate. The 10ppb standard came back as 9.8ppb. Finally the diluted Ag NP samples were determined followed by repeating the calibration standards at the end to make sure the instrument reading kept accurate.

### Cell culture and cytotoxicity assay

Human lung adenocarcinoma cell line (A549) [34], human embryonic kidney cell line (HEK293) [35] and human Liver cell line (HepG2) [36] were obtained from American Type Culture Collection (ATCC-LGC, UK) and cultured in Dulbecco's Modified Eagle Medium (DMEM) that were supplemented with 10% FBS and 100 U/ml of penicillin/streptomycin. Primary human dermal fibroblast cells (HDFc, C0135C) were purchased from Invitrogen [37] and cultured in DMEM/F12 media. Primary human coronary artery endothelial cells (hCAECs) [38] were purchased from PromoCell (Germany) and cultured in Endothelial growth media with supplements (PromoCell) in 75 cm² flasks in 5% CO_2_ incubator at 37°C.

MTT (3-(4,5-dimethylthiazol-2-yl)-2,5-diphenyltetrazolium bromide) assay was used to determine the toxicity of the laser generated Ag NPs to the five types human cells above. This assay is a colorimetric analysis which is based on the cleavage of tetrazolium salt by mitochondrial dehydrogenases that exist in the living cells to formazan with a purple colour. The amount of formazan product is proportional to the number of living cells in the culture and has invert correlation to the cell death. To determine the toxicity of the Ag NPs to the human cells, the cells were dissociation from the 75 cm² flasks using TrypL E (Sigma) and then seeded in 96 well-plates in a density of 50 cells / well for HEK293, A549 and HDF cells, and 100 cell / well for the HEMC-1 and HepG2 cells. Twelve hours after cell seeding, the culture media was replaced by 100 μl fresh media containing either 2.5 μg/ml or 25 μg/ml laser generated Ag NPs and incubated at 37°C in the 5% CO2 incubator for 24, 48 and 72 hours, respectively. Ten μl of MTT solution (5 mg/ml MTT in RPMI-1640) was added to each well and then 90 μl of culture medium were added to the same well to give a final MTT concentration of 10% (v/v). After 4 hours further culture at 37°C in the 5% CO_2_ incubator, 100 μl DMSO equivalent to the original culture volume) was added, and the plate was incubated for 30 minutes at room temperature with shaking for colour development. Finally the absorbance of the samples was read using a plate reader at a wavelength of 600 nm. The background absorbance was measured at wavelength 690 nm.

### Transmission Electron Microscopy

For the imaging of the A549 human lung cells, the cells were first seeded in a 10 cm Petri dish and incubated for overnight. Then the laser generated Ag NPs were added to the culture to give a final concentration 20 μg/ml and incubated at 37C° overnight. The cells were fixed with 4% formaldehyde containing 2.5% glutaraldehyde in 0.1M Hepes buffer (pH 7.2) for 1 hour. Then the cells were treated with 1% osmium tetroxide and 1.5% potassium ferrocynaide in 0.1M cacodylatebuffer (ph7.2) for 1 hour, followed by 1% tannic acid in 0.1M cacodylate buffer (pH 7.2) for 1 hour and finally in 1% uranyl acetate for 1 hour. The samples were dehydrated in ethanol series infiltrated with TAAB 812 resin and polymerized for 24h at 60°C. Sections were prepared with Reichert Ultracutultramicrotome and observed with FEI Tecnai 12 Biotwin microscope at 100kV accelerating voltage. The samples then analysed using the Bio Twin TEM (Hitachi H-7500, Germany).

For the imaging of *E*. *coli*, 1 ml of bacterial suspension (10^4^ cfu/ml) after overnight incubation were mixed with 500 μl fixative and left at room temperature for 1 hour. The cells were washed with cold PBS three times and fixed in 2.5% gulutaraldehyde for 12 hours and washed with PBS again three times and post-fixed in 1% osmium tetroxide for 4 hours. The samples then washed with PBS and dehydrated in series of acetone (50, 70 and 80%) and in 90% twice for duration of 10 minutes and each samples also washed in 100% acetone for 15 minutes twice. Finally, ultra thin sections were prepared for TEM images collected on copper grid and contrasted with uranyl acetate and lead strate. The samples then analysed using the Bio Twin TEM (Hitachi H-7500, Germany).

### Statistical analysis

Data in this study was presented as mean ± SE. A two-tailed Student’s t-test was conducted for all the data to evaluate the differences between samples. p ≤ 0.05 was considered as statistically significant.

## Results

### Laser generated Ag NPs have strong antibacterial activity against both gram-negative and gram-positive strains including MRSA

**Fig 1:**
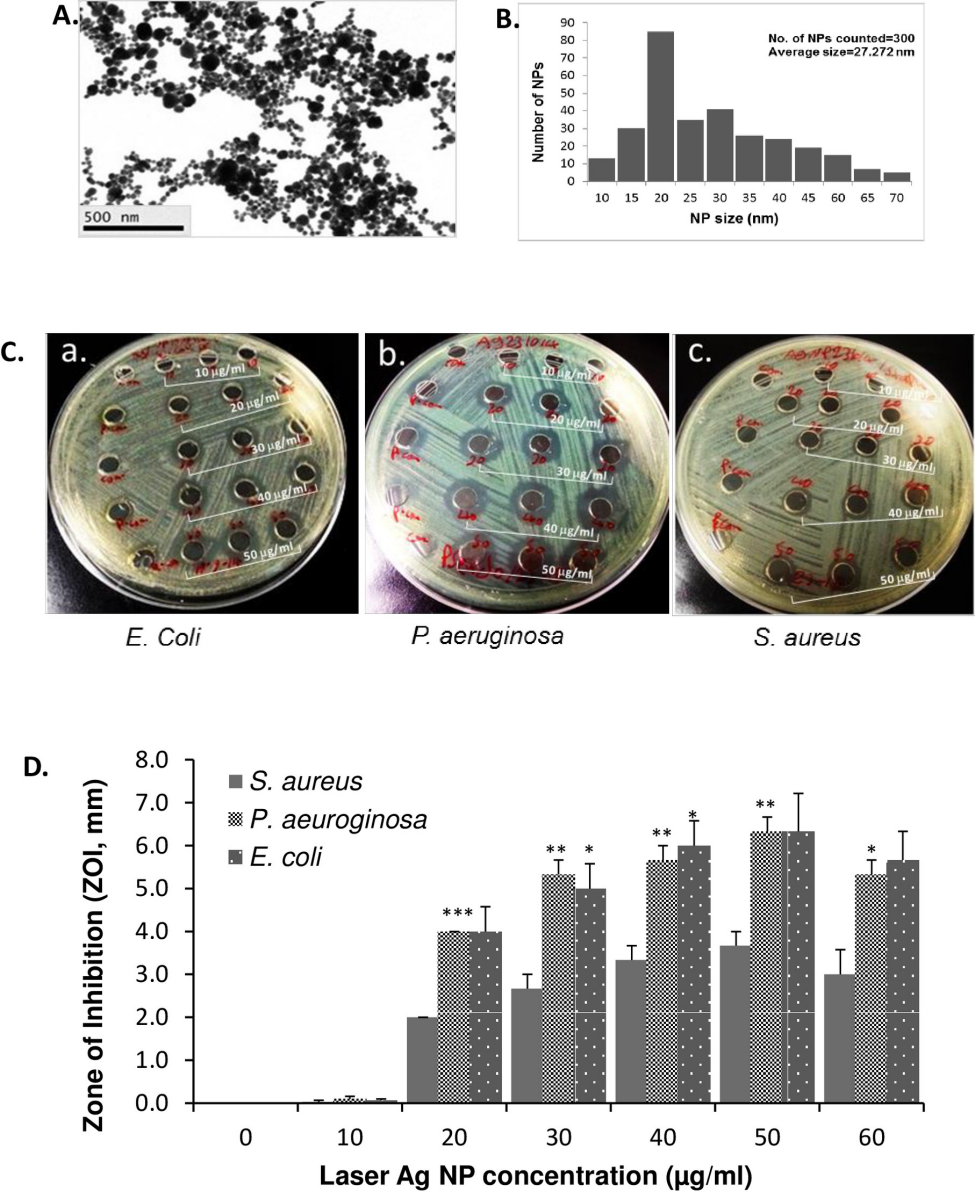
The antibacterial activity of laser generated Ag NPs against gram positive and negative bacteria. TEM Image of laser Ag NPs (**A.)**. Size distribution of laser Ag NPs (**B.**). A lawn of *E*. *coli*, *S*. *aureus* and *P*. *aeruginosa* were made on Muller-Hinton agar plates, respectively, and then 6 mm wells were created through the agar. Fifty μl of Ag NPs at different concentrations (0, 10, 20, 30, 40, 50 and 60 μg/ml) were added into each well in triplicate for each concentration, and the plates were incubated at 37°C for 24 hours. ZOI (**C.**) was measured and the readings were corrected by the diameter of the well (**D.**). Data are presented as mean ± SE. Compared to *S*. *aureus* at the same concentration of Ag NPs, *p ≤ 0.05, ** p ≤ 0.01 and *** ≤ 0.001, n = 3.

The average size of Ag NPs generated by picosecond laser is 27.2 nm, ranging 10–70 nm (Fig 1A and 1B). The antibacterial activity of the different concentrations of laser Ag NPs (10, 20, 30, 40, 50 and 60 μg/ml) was first tested against gram negative bacteria, *E*. *coli* and *P*. *aeruginosa*. Results showed that after co-incubation with bacteria at 37°C for 24 hours, the laser generated Ag NPs created clear ZOIs against *E*. *coli* which was in a concentration dependent manner (Fig 1C-a and 1D), indicating a significant antibacterial effect of the laser Ag NPs against *E*. *coli*. Similarly, a significant antibacterial activity of laser Ag NPs against *P*. *aeruginosa*, another example of gram negative bacteria, was observed in the same condition (Fig 1C-b and 1D). The maximum antibacterial effect of laser Ag NPs against both *E*. *coli* and *P*. *aeruginosa* was at a concentration 50 μg/ml (Fig 1C and 1D). The control wells that were loaded with sterile distilled water showed no ZOI.

The methicillin resistant *S*. *aureus* strain (MRSA) was used as an example of gram positive bacteria strains. Similarly, we observed dose-dependent antibacterial effect of the laser Ag NPs against MRSA, confirming the ability of laser Ag NPs in killing drug resistance strains. However, the ZOI for *S*. *aureus* was significantly smaller compared to that of *E*. *coli* and *P*. *aeruginosa* (Fig 1C and 1D). The different effect is likely caused by the structural differences between gram positive and gram negative bacteria [39], suggesting the bactericidal activity of laser generated Ag NPs depend not only on the concentration of NPs, but also the type of bacteria.

### Laser generated Ag NPs specifically induce the generation of non-hydroxyl ROS

**Fig 2:**
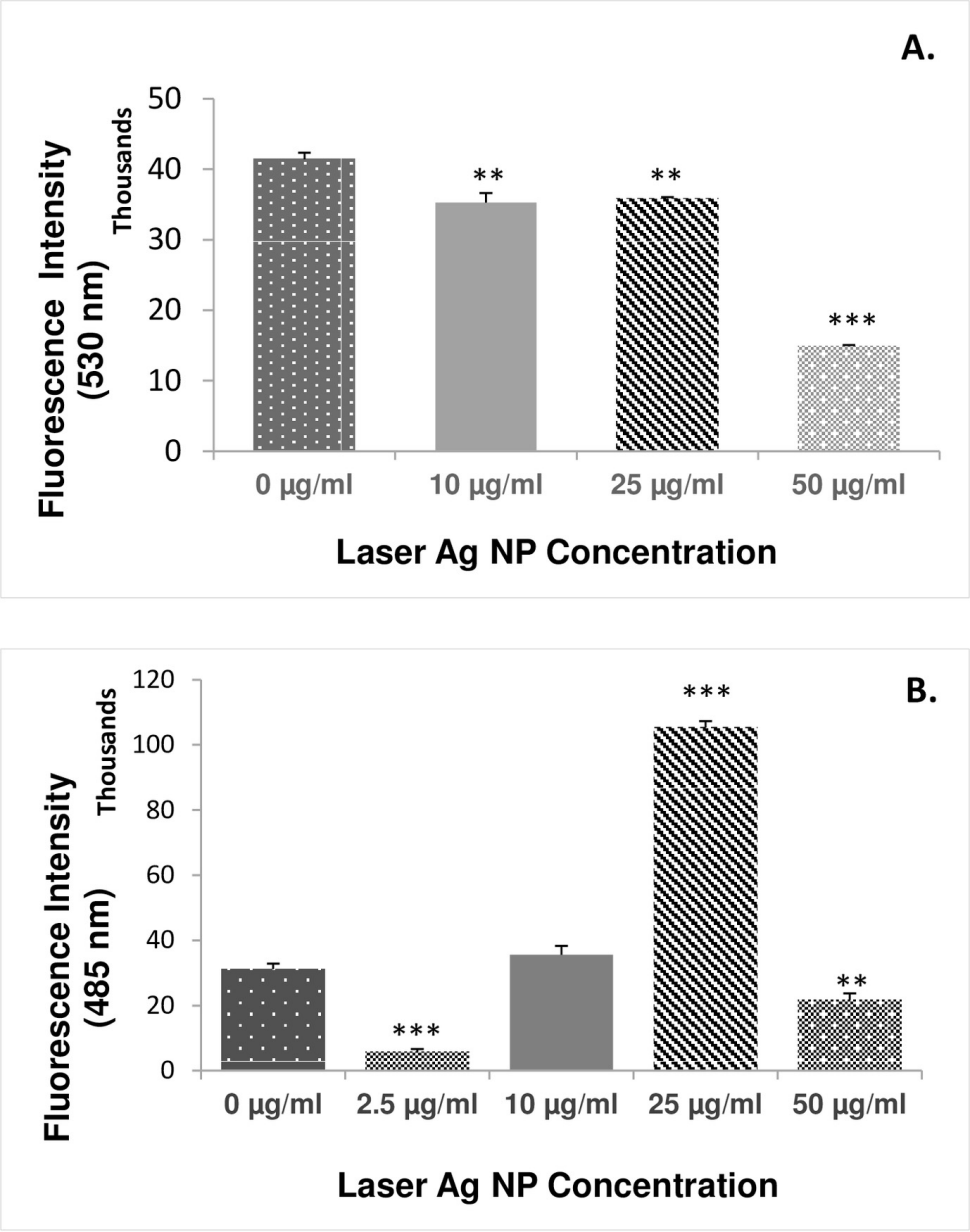
The effect of laser generated Ag NPs on the production of reactive oxygen species (ROS) in *E*. *coli*. Different concentrations of laser Ag NPs (10, 25 and 50 μg/ml) were cultured with *E*. *coli* for 5 hours in triplicate. The ROS levels were measured using the DCFH-DA kit (A.) and HPF kit (B.), respectively, and presented as the fluorescence intensity. Data are mean ± SE. Compared to the NP-free control, **p≤0.01, and ***p≤0.001, n = 3.

In order to elucidate the molecular mechanisms underlying the bactericidal effect of the laser generated Ag-NPs, ROS generation was measured using *E*. *coli* as a model system. Firstly, the fluorescein reagent HPF was used for the ROS measurement. After 5 hours co-incubation with the bacteria, the laser generated Ag NPs did not induce significant ROS generation at concentrations 10–50 μg/ml. In contrast, the level of ROS was slightly reduced at Ag NP concentrations of 10 and 25 μg/ml, and significantly decreased at the concentration 50 μg/ml (Fig 2A). As the HPF indicator is only specific to hydroxyl radicals, our results suggested that the hydroxyl radicals are unlikely to be the major mediators for the bactericidal effect of the laser generated Ag NPs.

We then used ROS indicator DCFH-DA which measures a wide spectrum of different ROS species including singlet oxygen, super oxide, hydrogen peroxide in addition to hydroxyl radicals (Fig 2B). Result showed that the laser generated Ag NPs at concentration 25 μg/ml induced highly significant ROS generation, about 3 folds higher than that of the non-NP control sample (Fig 2B), suggesting the presence of significant oxidative stress. The differential ROS generation as determined by HPF and DCFH-DA reagents demonstrated for the first time that laser Ag NPs specifically induce the formation of non-hydroxyl radicals.

**Fig 3:**
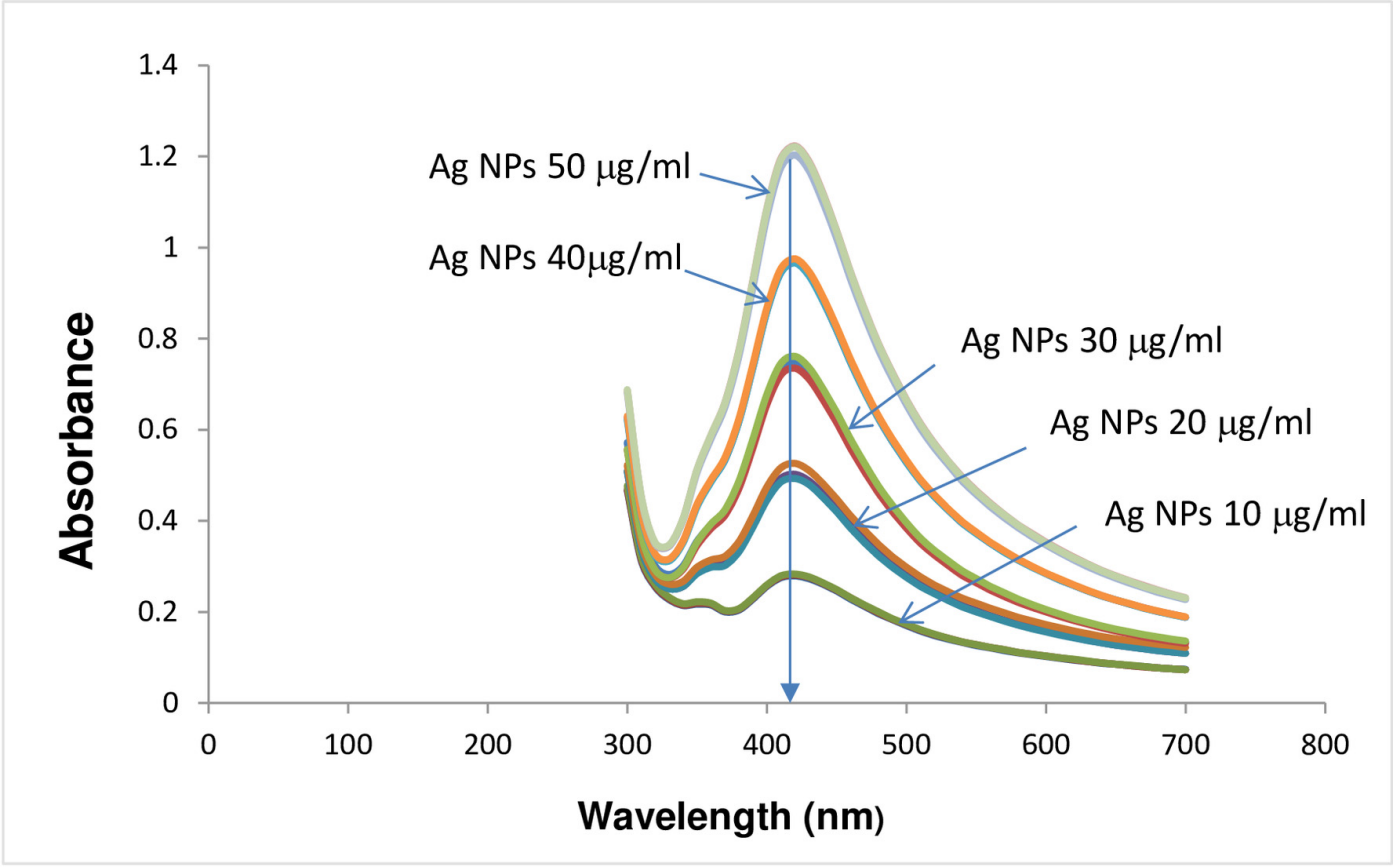
Absorbent spectrum of laser generated Ag NPs. The absorbent spectrum of different concentrations of laser generated Ag NPs was measured by UV-VIS Spectrophotometer. Triplicate samples were measured at each Ag NP concentration.

It is interesting to note that the level of ROS generation was significantly lower than the control samples when higher concentration of Ag NPs (50 μg/ml) was used. The decreased ROS level at high NP concentrations agreed with the results by Kaur et al [40] where it was partially explained as the aggregation of NPs at high concentrations which reduces the number of NPs available to interact with the bacteria. However, we did not detect any aggregation tendency for the laser Ag NPs at higher concentrations as evidenced by the similar peak wavelengths determined by UV-visible spectrometer (Fig 3) and the zeta potentials (Table 1) among samples with different Ag NP concentrations. The average zeta potentials for all concentrations of Ag NPs tested were less than—25 mV, suggesting a dispersion stability of the laser Ag NPs.

**Table 1:** Zeta potential of laser Ag NPs.

Ag NP Concentration(μg/ml)	Zeta Potential (mV)(mean ± St Dev)
10	-38.3 ± 8.58
20	-33.5 ± 8.30
30	-34.2 ± 7.49
40	-37.7 ± 9.54
50	-35.9 ± 8.07

### Laser generated Ag NPs cause glutathione depletion in bacterial cells

**Fig 4:**
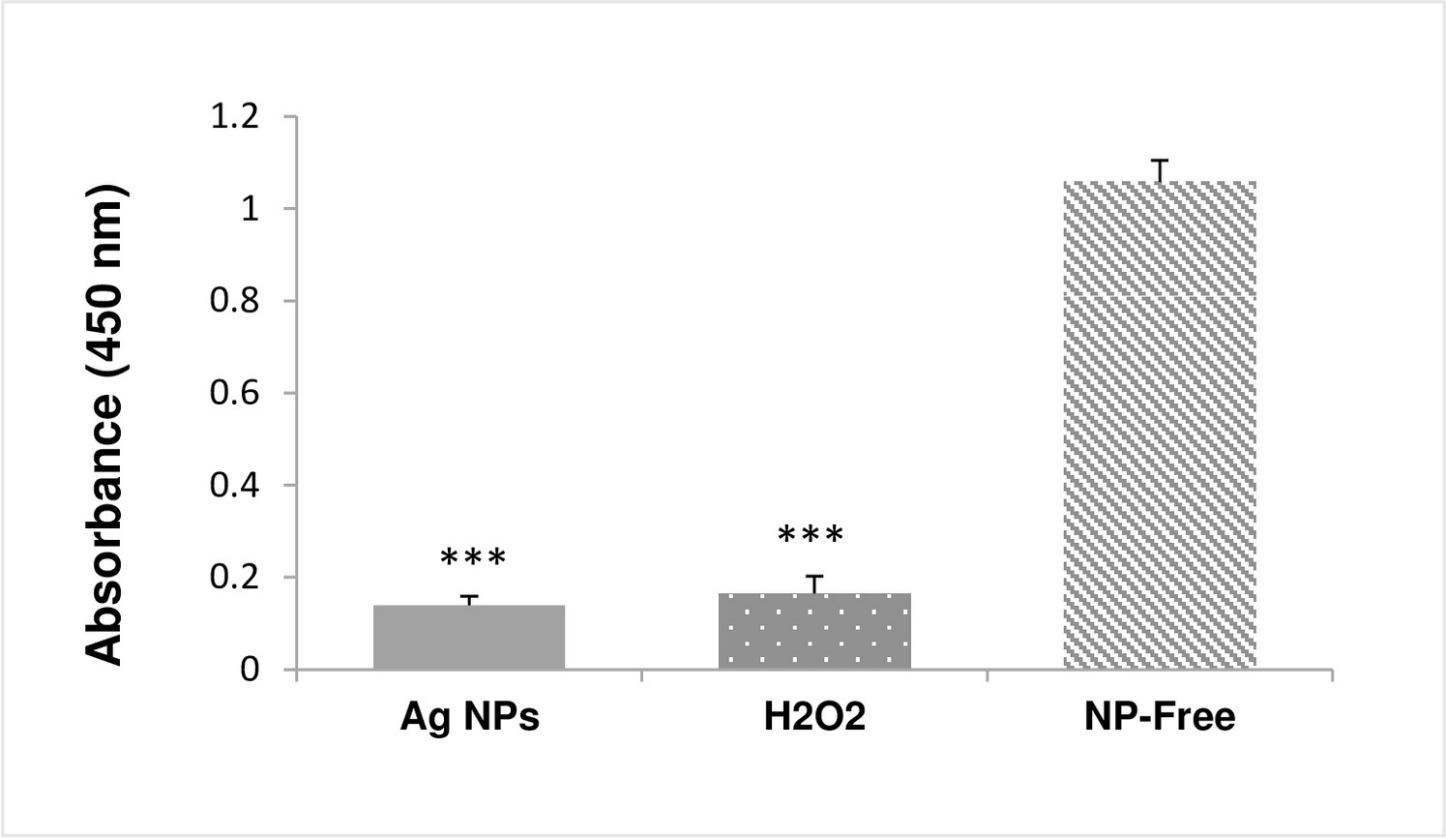
Changes of cellular glutathione level in laser Ag NP treated *E*. *coli*. *E*. *coli* were treated with laser Ag NPs (15 μg/ml) for 3 hours. The cellular glutathione level was measured using the glutathione assay kit (Sigma). H_2_O_2_ (4 μg/ml) treatment was used as a positive control, and the NP-free sample as a negative control. Data are presented as mean ± SE. Compared to the NP-free control, ***p≤0.001, n = 3.

Glutathione reductase catalyses the formation of glutathione (GSH) that is a critical antioxidant to prevent cellular damages by oxidative stress. Such defence mechanisms could likely be exhausted due to the accumulation of ROS, resulting in depletion of cellular GSH leading to oxidative stress-induced cell death. To determine if this is a potential molecular mechanisms that contributes to the bactericidal effect by the laser generated Ag NPs, the level of GSH in *E*. *Coli* was measured after the bacteria been exposed to the laser generated Ag NPs (15 μg/ml) for 3 hours. Results showed that the glutathione reductase level was significantly lower than that of the non-laser Ag NPs treated control sample (Fig 4), suggesting cellular depletion of GSH happened to the laser Ag NPs treated *E*. *Coli*.

### Laser generated Ag NPs induce bacterial lipid peroxidation

**Fig 5:**
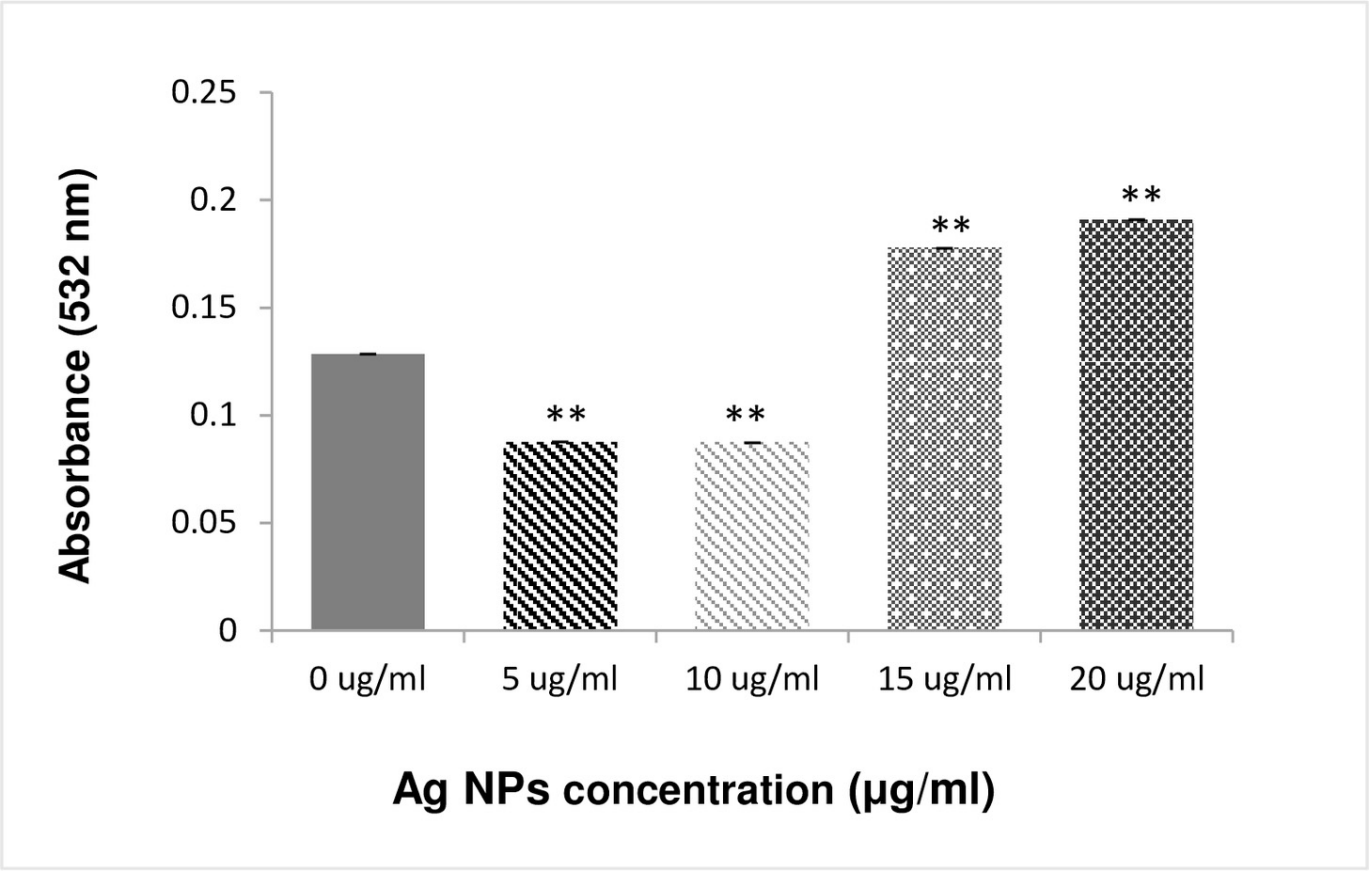
Lipid peroxidation in E. coli by laser generated Ag NPs. The bacterial cells were treated with different concentrations of laser Ag NPs for 3 hours. Cellular level of malondialdehyde was then measured using the MDA assay Kit (Sigma-Aldrich). Data are presented as mean ± SE. Compared to the NP-free control, **p≤0.01, n = 3.

Cellular accumulation of ROS leads to lipid peroxidation (LPO), a key mechanism responsible for the increase in cell membrane permeability that contributes to cell death. The generation of malonealdehyde (MAD) is a useful marker to monitor LPO. To determine if the increase in ROS generation by laser generated Ag NPs correlates to the increase of LPO in the bacterial cells, we measured the cellular production of MAD. Fig 5 showed that the MAD level was significantly increased in the *E*.*Coli* after the exposure to the laser Ag NPs at concentrations 20 μg/ml.

### Laser generated Ag NPs reduce the integrity of the bacterial cell membrane

**Fig 6:**
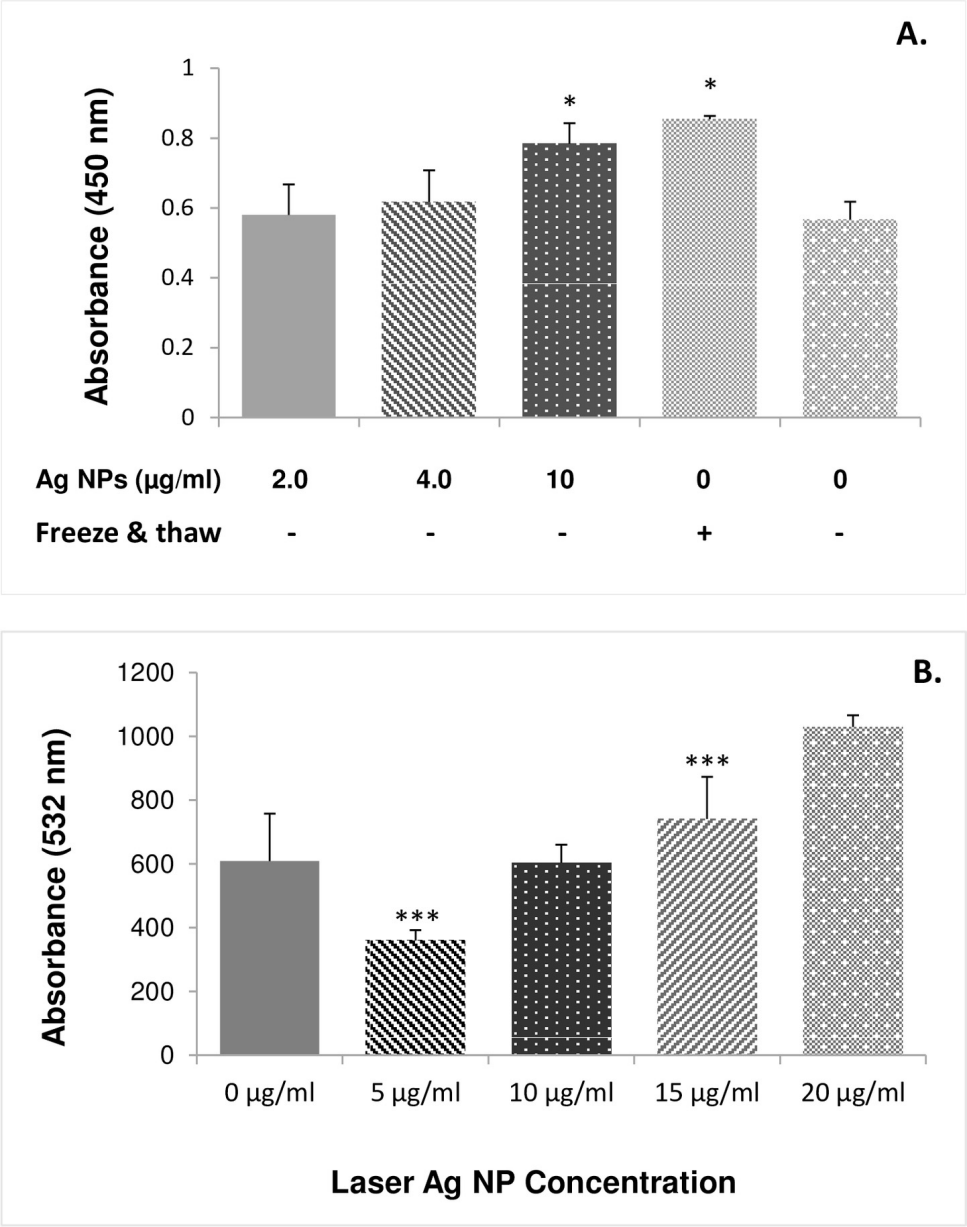
Effect of laser generated Ag NPs on E. coli membrane integrity. The *E*. *coli* were treated with different concentrations of laser Ag NPs for 24 hours. The culture media were subject for cell membrane integrity analysis using either the LDH assay kit (A.) or the Coomassie reagent (B.), respectively. A positive control was used for the LDH method by freezing and thawing of the E. coli culture to physically break up the cell membrane integrity. Data are mean ± SE. Compared to the NP-free control, **p≤0.01, and ***p≤0.001, n = 3.

We next determined the bacterial cell membrane integrity after being exposed to laser Ag NPs. LDH release is considered to be a reliable indicator of cell membrane damage and permeability increase. When *E*. *coli* were exposed to 2.0 or 4.0 μg/ml laser Ag NPs for 24 hours, we did not detect LDH increase in the culture media (Fig 6A). However, when the concentration of Ag NPs increased to 10 μg/ml, a significant LDH release was detected (Fig 6A), suggesting cell membrane damage had occurred.

The loss of cell integrity was also demonstrated using protein leakage analysis. After treating *E*. *coli* bacterial culture with different concentrations of laser Ag NPs (5, 10, 15 and 20 μg/ml) for 24 hours, a dose dependent increase of the protein level was detected in the culture media as compared to the NP-free control sample (Fig 6B).

### Laser generated Ag NPs release Ag ion

The ionic form of silver (Ag^+^) is considered to be one of the key mechanisms for the bactericidal effect of Ag NPs. We accurately measured the amount of Ag^+^ release from the Ag NPs in deionised H_2_O using ICP-MS. As shown in Table 2, a significant amount of Ag^+^ was detected in the Ag NP solution. The amount of Ag^+^ was increased from 1838.993 μg/L to 3309.023 μg/L when the Ag NP concentration increased from 20 μg/ml to 50 μg/ml (Table 2).

**Table 2:** Silver ion concentration in Ag NP samples.

Ag NPs	Silver ion (μg/L)
Laser Ag NPss (20 μg/ml)	1838.993
Laser Ag NPs (50 μg/ml)	3309.023
Commercial Ag NPs (20 μg/ml)	17895.760

We also measured the Ag^+^ concentration for the commercial Ag NPs (Sigma) that was made by chemical synthesis. To our surprise, the chemically made Ag NPs had about 10-fold higher Ag^+^ concentration than that of the laser Ag NPs. Interestingly, the chemically made Ag NPs did not show any superior antibacterial activity (ZOI 5.5 ± 0.5 mm) than that of laser generated Ag NPs (ZOI 5.1 ± 1.1 mm, p>0.05) when the same concentration of Ag NPs were used (20 μg/ml), suggesting other mechanisms addition to Ag+ play an important role in the antibacterial effect by laser generated Ag NPs.

### Laser generated Ag NPs penetrate into bacterial cells

**Fig 7:**
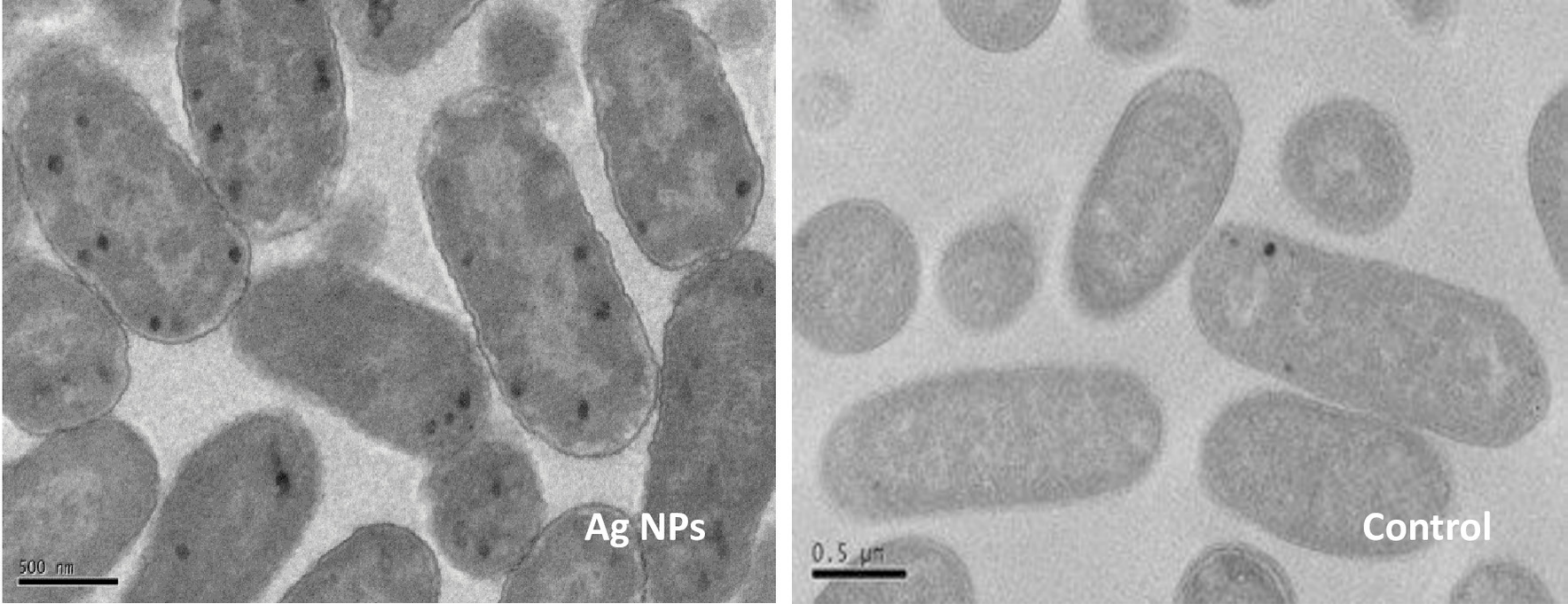
TEM images of laser generated Ag NPs penetrating into *E*. *coli*. TEM imaging was conducted on *E*. *coli* cells that were treated with laser Ag NPs (50 μg/ml) or without Ag NPs (Control) for 24 hours. Scale bar: 0.5μm.

To observe the interaction of the laser Ag NPs with bacterial cells, TEM imaging was performed on *E*. *Coli* after been cultured overnight with the laser generated Ag NPs. Fig 7 shows that considerable amounts of laser Ag NPs were penetrated the bacterial cell membranes and entered the cells. Compared to the NP-free control cells, the membranes of NP-treated *E*.*coli* had irregular appearance and the cell body became obviously swollen (Fig 7), further supporting the increased cell membrane permeability caused by laser Ag NPs.

**Fig 8:**
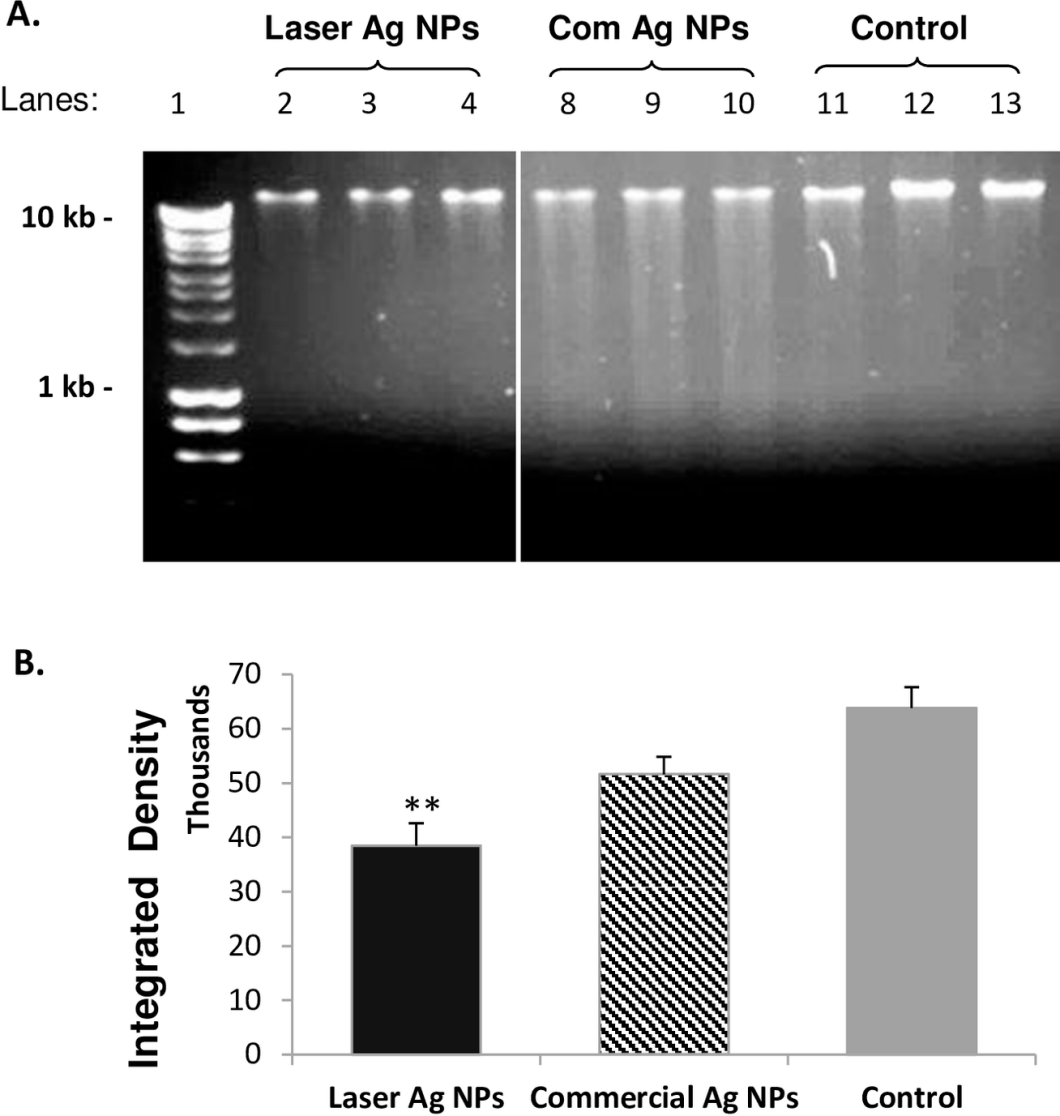
DNA degradation by laser Ag NPs in *E*. *coli*. *E*. *coli* were treated by laser generated Ag NPs (10 μg/ml) or commercial Ag NPs (4 μg/ml) for overnight. Genomic DNA were extracted and 200 ng DNA were subjected to agarose gel electrophoresis (A). The intensity of the major DNA bands were quantified and shown in (B). Lane 1, Hyper ladder I (BioLine); Lane 2–4, laser Ag NPs; Lane 8–10, Commercial Ag NPs; Lane 11–13, NP-free Control. Data were presented as mean ± SE. Compared to the NP-free control, **p≤0.01, n = 3.

The effect of laser Ag NPs on *E*. *coli* DNA damage was then determined using DNA agarose gel electrophoresis. By loading the same amount of DNA (200 ng/well), the density of the major band that indicates the intact genomic DNA of *E*.*coli* was significantly reduced in the DNA samples extracted from the laser Ag NPs treated *E*.*coli* than that of the NP-free control (Fig 8), indicating the laser generated Ag NPs had induced DNA degradation. However, we did not observe obvious DNA laddering for the laser Ag NP treated samples (Fig 8 lanes 2–4), suggesting that a rather sever DNA damage occurred where the small fragments of degraded DNAs had run out of the visible range of the agarose gel. This was in contrast to the DNA samples extracted from *E*.*coli* treated with the chemically made commercial Ag NPs where obvious DNA laddering was observed (Fig 8 lanes 8–10). This was likely due to the less amount of commercial Ag NPs (4 μg/ml) were used in the experiment which generated larger sized DNA fragments indicating less extend of DNA degradation compared to that of the laser Ag NP-treated samples.

### The cytotoxicity of laser generated Ag NPs to human cells

The cytotoxicities of laser Ag NPs against five types of different human cells originated from the lung (A549 line), the blood vessel (hCAECs), the kidney (HEK293 line), the skin (HDFc cells) and the liver cells (HepG2 line) were evaluated. The cells were exposed to the laser Ag NPs for 3 different time periods (24, 48 and 72 hours) at both a low (2.5 μg/ml) and a relatively high (20 μg/ml) NP concentrations followed by MTT assay to measure the viable cells which inversely correlated to the number of dead cells.

**Fig 9:**
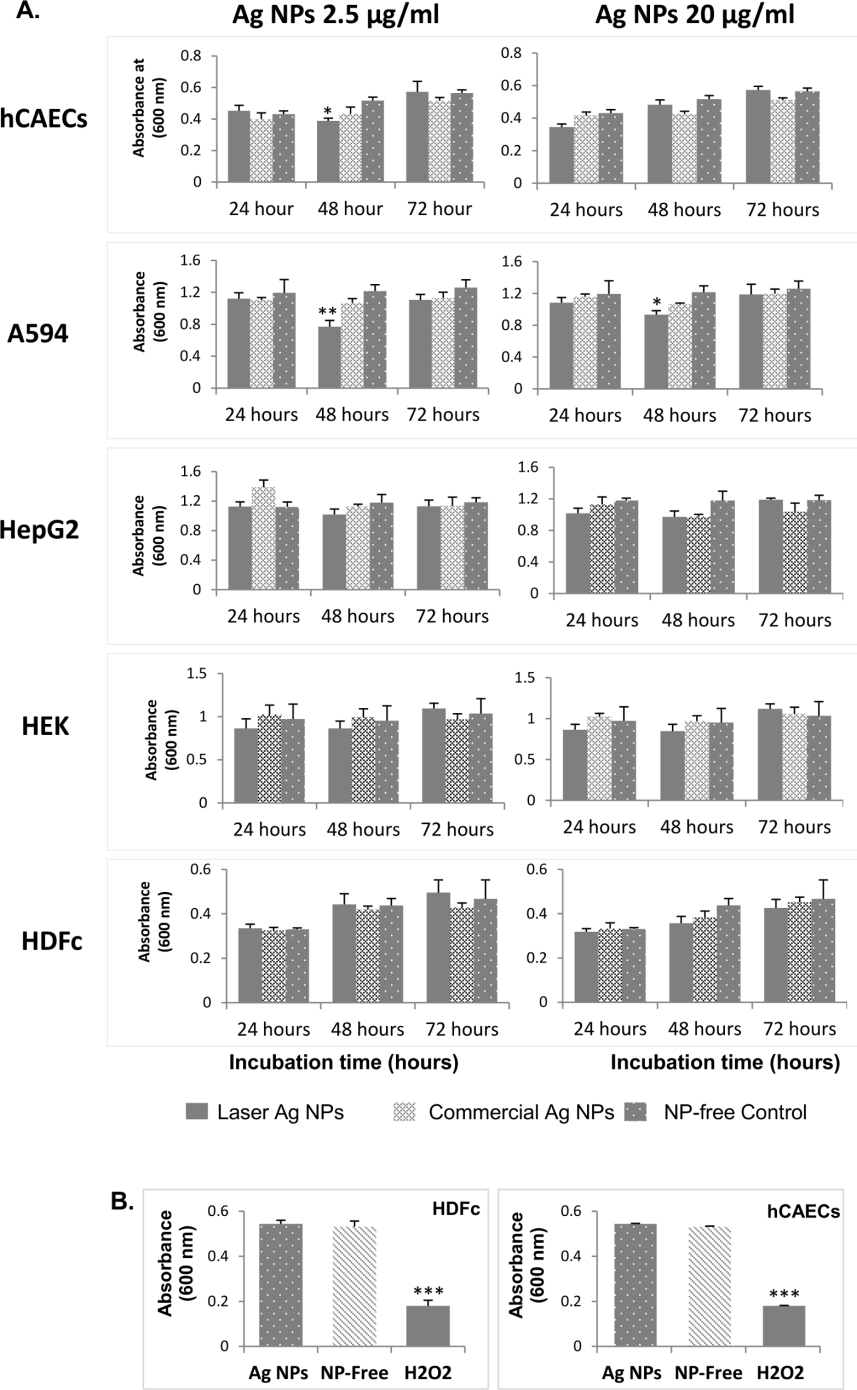
Cytotoxicity of laser generated Ag NPs to human cells. The laser generated Ag NPs at two concentrations (2.5 and 20 μg/ml) were incubated with five types of human cells or cell lines: A549 (lung), hCAECs (blood vessel), HEK293 (kidney), HDFc (skin) and the HepG2 (liver), for 24, 48 and 72 hours, respectively. The cytotoxicity was determined using MTT assay (A). H_2_O_2_ (40 μg/ml) treatments of HDFc and hCAECs, alongside with 20 μg/ml Ag NP treatments, for 24 hours were used as positive controls (B). Data were presented as mean ± SE. Compared to the NP-free control, *p≤0.05, **p≤0.01, ***p≤0.001, n = 3.

After 48 hours exposure, the A549 cells exhibited mild but statistically significant cell death at both NP concentrations, while the viability of the endothelial cells were only affected at laser Ag NPs concentration 2.5 μg/ml (Fig 9A). Other cell types did not have significant cell death at this time point. Interestingly after 72 hours incubation, the growth of A594 cells and endothelial cells had caught up with other cell types. The laser generated Ag NPs did not induce any significant cell death to all cell types tested (Fig 9A), suggesting a low toxicity of the laser generated Ag NPs to human cells at this time point.

To verify the reliability of the cytotoxicity assay by demonstrating the cytotoxicity can be readily detected by the MTT assay, hydrogen peroxide (H_2_O_2_), a known ROS species with high toxicity to living cells, was used as a positive control. When HDFc and hCAECs cells were treated with H_2_O_2_ (40 μg/ml), significant cell death were observed on both cell types as compared to the control, while no significant toxicity was seen on cells treated by 20 μg/ml laser Ag NPs (Fig 9B). This experiment has therefore validated the MTT assay used in our study.

**Fig 10:**
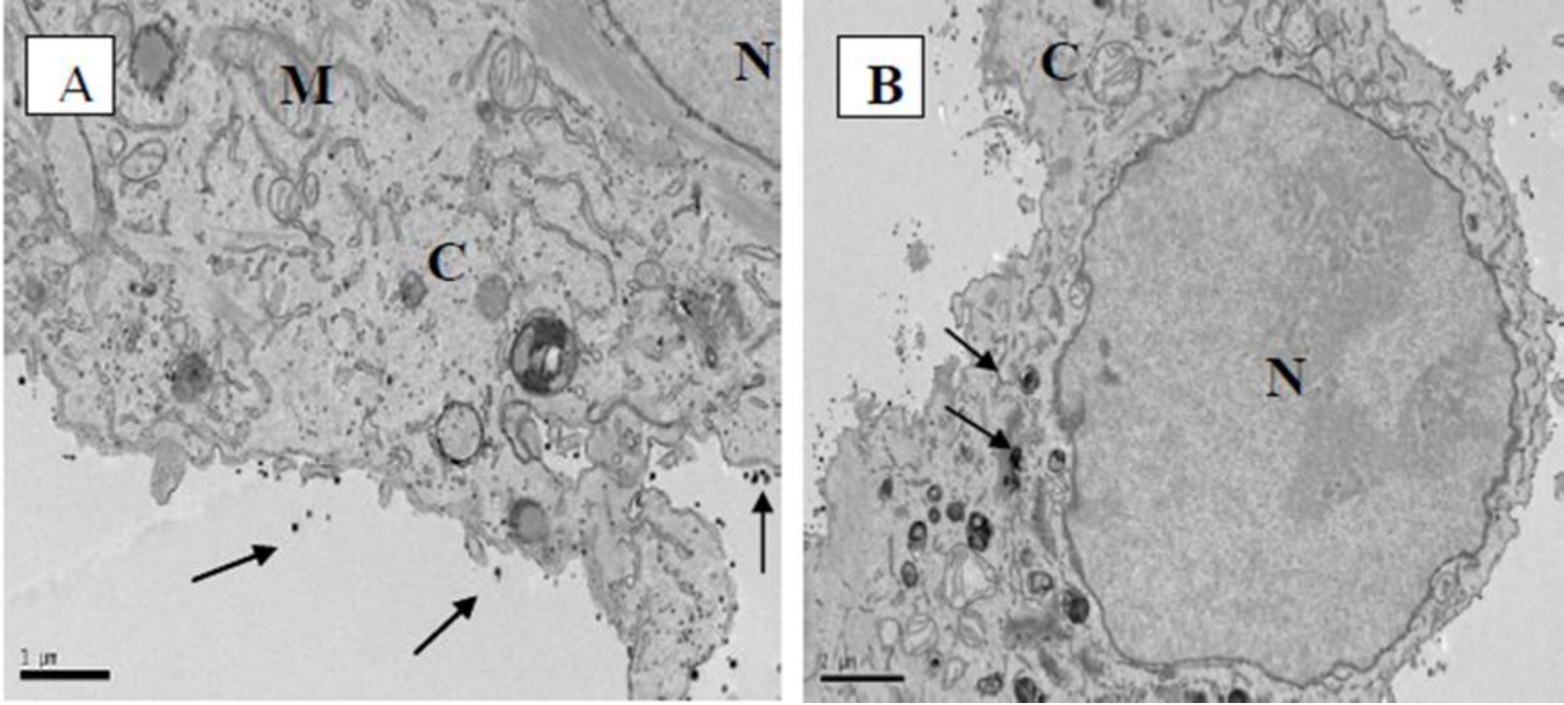
TEM images of human lung AC549 cell line treated with laser generated Ag NPs. TEM imaging was conducted on A549 cells that were treated with laser generated Ag NPs (20 μg/ml) for 24 hours. The arrows in (A) indicate the attachment of Ag NPs to the cell membrane. The arrows in (B) indicate the vesicular structure containing Ag NPs. C, cytoplasm; N, nucleus; M, mitochondria.

To examine more closely the interaction of laser Ag NPs with human cells, TEM was used to visualise Ag NP treated A594 cells. After co-culturing A594 cells with laser generated Ag NPs (20 μg/ml) for 24 hours, the Ag NPs were mainly attached to the cell membranes (Fig 10A) with a small amount seen inside the cells (Fig 10B). The intracellular Ag NPs were surrounded by organelle membranes (or by vacuoles). We did not observe any Ag NPs in the cell nuclei, suggesting the low DNA toxicity to human cells.

## Discussion

### Antibacterial activities of laser generated Ag NPs

In this study, the antibacterial activities of laser Ag NPs against both gram negative (*E*. *coli* and *P*. *aeruginosa*) and gram positive (*S*. *aureus*) bacteria were evaluated. The effects displayed significant dose dependency with Ag NP concentrations ranging from 10 μg/ml to 50 μg/ml (Fig 1). This is in line with other publications on the bacterial killing effects of Ag NPs in general including the work by Dror-Ehre et al, where it showed that the bactericidal activity of Ag NPs against *E*. *coli* relied on the ratio of NPs-cells which determines the frequency of collisions and attractions of the NP to the cells [41]. We also observed high susceptibilities of *E*. *coli* and *P*. *aeruginosa* to the laser generated Ag NPs as compared to that of *S*. *aureus* (Fig 1). This may be explained by the structural differences between gram positive and gram negative bacteria. For the gram positive bacteria, the peptidoglycan layer is thicker than gram negative bacteria which serve as a protective barrier against chemicals, toxins, derivative enzymes and antibiotics [42]. Importantly, in our study we have confirmed that the laser generated Ag NPs could effectively kill the methicillin resistant *S*. *aureus*, which provide good evidence for the future applications of laser generated Ag NPs to be used in healthcare in combatting drug-resistant microbial infection or contaminations.

In this study, the high power and high repetition rate picosecond laser was used for the production of Ag NPs. It has been shown that high repetition rate picosecond (ps) lasers are advantageous compared to femtosecond (fs) lasers if the total thermal load produced by laser irradiation can be redistributed across a larger area [10]. In case of laser ablation in a liquid flow, the thermal energy is dissipated into the liquid and drained by the flow. It has been shown that laser ablation in liquid produces surface-charged nanoparticles with a shell of dipole molecules (e.g., water) formed around them, preventing agglomeration [2]. This was experimentally supported by the zeta potential value of laser Ag NPs (Table 1).

### The molecular mechanisms for the antibacterial effects by laser generated Ag NPs

ROS generation is one of the key molecular mechanisms underlying bactericidal effects of NPs. To confirm the same mechanisms was used by the laser generated Ag NPs, ROS was measured by two types of different ROS indicators, Hydroxyphenyl fluorescein (HPF), the hydroxyl radical and peroxynitrite sensor, and 2,7-dichlorofluorescein diacetate (DCFH-DA) which has a broad detection spectrum. Based on the differential detection capability of the chemicals indicators, we dissected, for the first time, that the ROS generation induced by the laser generated Ag NPs did not induce detectable hydroxyl radical (·OH) (Fig 2). Our finding contradicts a publication by Hwang et al where silver nanoparticles could transmute H_2_O_2_ into hydroxyl radical leading to apoptosis in fungal cells [43]. However, our result agrees with a report by He et al., in which Ag NPs produced a powerful oxidant through a reaction with H_2_O_2,_ and the oxidizing species did not include the free hydroxyl radical [44]. Hydroxyl radical is highly reactive and can damage virtually all types of macromolecules, especially the nucleic acid and lipids [45]. The lack of significant generation of hydroxyl radicals but large amount of other ROS species by the laser generated Ag NPs in our experiment suggest that the molecular mechanisms underlying the antibacterial effect might depend on the method used for the nanoparticle production and the target microbial species. It would be interesting in a future study to compare the generation of specific ROS species induced by Ag NPs that are manufactured by different technologies. This will further clarify the specific properties of the Ag NPs that are generated by laser ablation.

We could not explain the low ROS levels when higher concentrations of (50 μg/ml) laser Ag NPs were present in the *E*.*coli* culture. Zeta potential is one of the fundamental parameters known to reflect the stability of the colloidal dispersions by measuring the magnitude of the electrostatic repulsion or attraction between particles. The zeta potential values (Table 1) of the laser Ag NPs samples suggested a low aggregation tendency for Ag NPs tested at all concentrations. One possible explanation for the low ROS level at high Ag NP concentration could be the significant cell death which may have influenced the ROS measurement. More work is needed to clarify this phenomenon in the future.

ROS activates the cellular antioxidant defence system in order to maintain equilibrium balance in the redox system. The excess accumulation of ROS will eventually deplete the cellular GSH pool leading to insufficient antioxidants to overcome the accumulated ROS, resulting in cell damage [46]. We found that the laser generated Ag NPs caused a highly significant reduction in GSH compared to that of the control *E*. *coli* (Fig 4), suggesting GSH depletion could be one of the major mechanisms underlying the antibacterial effects of the laser Ag NPs.

Bacterial cell membranes are considered to be the first barrier against ROS attachment. The poly unsaturated phospholipids are the main elements in bacterial cell membrane components. Damage to the membrane not only leads to the leakage of intracellular components, but also allows NPs to enter the cell to interact with essential enzymes in the bacterial respiratory chains, leading to respiratory inhibition and further ROS generation, contributing to cell death. MDA is a highly reactive by-product of lipid oxidation which has the ability to react with proteins and form a protein-MDA complex that is considered as a mutagenic compound. Our results showed that laser Ag NPs exposure to *E*. *Coli* has increased the production of MDA (Fig 5), which likely contributed to the disintegration of the bacterial cell membrane. This, together with the physical interactions between NPs and the bacteria, leads to an increased permeability in the cells as evidenced by the LDH and protein leakages (Figs 5 and 6), resulting in eventual cell death.

The observation of similar antibacterial effects but very low levels of Ag^+^ released for the laser Ag NPs as compared to the chemically generated Ag NPs suggest that the Ag^+^ ion may not be as important for the laser Ag NPs to kill bacteria as for the chemical Ag NP. This is an interesting phenomenon. It would be important for future experiments to dissect the contribution of Ag^+^ to the overall antibacterial effect of the laser generated Ag NPs in order to identify novel applications for this type of Ag NPs.

### The toxicities of laser Ag NPs to human cells

Generally, we observed low toxicity in the laser Ag NPs to the five different types of human cells, except for the lung and endothelial cells where weak but significant influences on cell survival by the NPs were detected (Fig 9A).

The endothelial cells that line the blood vessels are highly important as the first barrier for the NPs uptake [47]. The toxic effect of the laser Ag NPs was observed at 48 hours after the endothelial cells had received a low concentration (2.5 μg/ml) of the Ag NPs (Fig 9A). This could be an adaptive effect since following a further Ag NP challenge of up to 72 hours the cell viability was equivalent to the control cells. However, the lack of toxicity at 72 hours could also be due to the uptake saturation of the NPs by the cells [48].

The human respiratory system is considered to be the first organ that could be affected by exposure to NPs via inhalation, while both the cardiovascular system and the nervous system are considered as secondary organs which could be affected by NPs [49]. In this study, the A549 cell line was sensitive to the laser generated Ag NP treatment at both low (2.5 μg/ml) and high (20μg/ml) concentrations of laser Ag NPs (Fig 9A). However, similar to what we have observed on the endothelial cells, the toxic effects were no longer present when the NPs were further incubated with the A549 cells for up to 72 hours (Fig 9A). The TEM images of the A549 cells showed that the laser Ag NPs had the ability to enter the cells but almost all of the intracellular Ag NPs were located in the cytoplasm, not the nucleus. This may partially explain the mild cytotoxicity of the laser Ag NPs to human cells but significant bactericidal effect to microbes where the Ag NPs could be in direct contact with bacterial DNA in the cytosol upon entering the cells. This supports the previous findings by Xu et al [50] where they showed that NPs locating in the nuclei or ribosome of the cells are more toxic than those in the cytoplasm, even at low concentrations. Nevertheless, as compared to other cell types, our results indicate that the lung cells seem relatively more susceptible to the laser generated Ag NP treatment.

The lack of significant toxic effects on the majority of cell types and conditions employed in our experiment prompt us to evaluate the reliability of our MTT assay. Using H_2_O_2_ as a cytotoxic inducer, we successfully detected cell death for the H_2_O_2_ treated endothelial cells and dermal fibroblasts, while no significant cell death was observed when the same population of cells were treated by the laser Ag NPs (Fig 9B), confirming the robustness of our assays.

We did not find any significant toxic impacts of the laser Ag NPs to the kidney HEK293 cells, liver HepG2 cells and the skin fibroblasts. The lack of toxicity to the skin cells assures the safe usage of laser Ag NPs in treatment of wounds such as by being embedded in a bandage or wound dressing.

The size of NPs is considered to be critical determinant of their toxicity to both human cells and bacteria. It was noticed that NPs of a smaller size were more toxic than larger ones. This is partially because smaller sized NPs are more easily able to enter mitochondria and cause oxidative stress by stimulating ROS generation, which leads to cell death through apoptosis [51]. The laser Ag NPs used in our study, although clean, have a size range of 10–100 nm with an average of 27.272 nm. A further study we will seek to carry out will separate the different sized NPs via fractionation to more precisely determine their correlation to their biological function and toxicity.

In this study we have also compared the toxicity of laser Ag NPs to chemically prepared NPs (commercial Ag NPs) to human cells. Overall, we did not observe significant differences of the human cell toxicity between the two types of Ag NPs. The size of the commercial Ag particles we used was 40 nm. The length of storage time for the commercial Ag NPs was also different from the laser Ag NPs we prepared, which may have an impact on the activity of NPs. For precise comparison, the NP size and shelf live should be taken into account in our future study.

## Conclusions

Silver NPs generated by a picosecond laser have strong antibacterial effects against both gram negative and gram positive strains including MRSA. The toxicity of laser Ag NPs to human cells are minimal based on the *in vitro* test on human cells models within 72 hours. The bactericidal effects are mediated by oxidative stress, likely via the mechanisms of lipid peroxidation and depletion of glutathione leading to disintegration of cell membrane and DNA damage.
