# Correction: The Molecular Mechanisms of the Antibacterial Effect of Picosecond Laser Generated Silver Nanoparticles and Their Toxicity to Human Cells

**DOI:** 10.1371/journal.pone.0203636

**Published:** 2018-08-30

**Authors:** Peri Korshed, Lin Li, Zhu Liu, Tao Wang

Fig 7 showed TEM images of laser generated silver nanoparticles (Ag NPs) penetrating *E*. *coli*. The authors recently noticed that these black dots might not be the actual nanoparticles as similar dots were also found in the control samples. To verify the results the experiment was repeated, which produced much clearer and convincing images of the Ag NPs. The new images showed that the Ag NPs did penetrate *E*. *coli* as shown in the original figure. The authors have provided a corrected version here.

**Fig 7:**
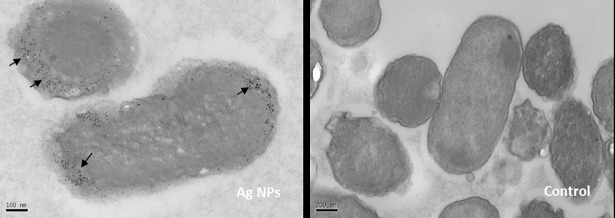
TEM images of laser generated Ag NPs penetrating into *E*. *coli*. TEM imaging was conducted on *E*. *coli* cells that were treated with laser Ag NPs (50 μg/ml) or without Ag NPs (Control) for 24 hours.
